# Histones deacetylases in the epidermis: structure, functions and therapeutic implications

**DOI:** 10.3389/freae.2025.1711733

**Published:** 2025-12-08

**Authors:** Chloé Nguyen Van, Jérôme Lamartine

**Affiliations:** Laboratory of Tissue Biology and Therapeutic Engineering (LBTI), CNRS UMR5305 – University Claude Bernard Lyon I, Lyon, France

**Keywords:** HDACs, epidermis, skin, epigenetics, melanoma, psoriasis

## Abstract

Histone deacetylases (HDACs) are key epigenetic regulators that catalyze the removal of acetyl groups from histones and non-histone proteins, thereby modulating chromatin accessibility and gene expression. The HDAC family is composed of 11 HDACs and seven sirtuins that are all located within the epidermis. These enzymes are involved in essential cellular processes such as proliferation, differentiation, the regulation of immune function and wound healing, thus playing a central role in maintaining tissue homeostasis. This review aims to explore the mechanistic contributions of HDACs to epidermis physiology and investigate their involvement in the development of diseases such as psoriasis, atopic dermatitis and cancers. It also highlights the therapeutic potential of HDAC inhibitors, which are already used in oncology but whose application in dermatology is still emerging.

## 1 Introduction

Skin is a complex organ with multiple functions. The barrier function of the skin, mainly ensured by the epidermis, is essential to protect the body against external aggressions and to limit internal water loss. Rupture of this barrier, due to deep and extensive wounds, can be life-threatening. The epidermis is mostly composed of 90% keratinocytes but also comprises melanocytes responsible for the pigmentation of skin and hair, Merkel cells involved in mechano-perception, as well as Langerhans cells and other resident immune cells such as T lymphocytes that ensure innate cutaneous immunity. Epidermal homeostasis is maintained by complex processes that enable its continuous renewal throughout life, and the maintenance of correct interactions between the different cell types that make it up - interactions that are essential for epidermal functions. These multiple processes involve fine gene regulations, which control the main cellular mechanisms such as proliferation, differentiation, migration or cell death. The regulation of gene expression is performed at both transcriptional and post-transcriptional levels, two complementary steps in time and space. The basic transcriptional control involves DNA regulatory sequences on which regulatory proteins can bind and then activates or repress gene transcription. The post-transcriptional level includes additional steps such as the control of mRNA stability, splicing, and translation. Regulation of the chromatin compaction rate, and therefore the local accessibility of DNA to specific regulatory proteins, is a higher level of transcriptional regulation involving specific chemical modification in basic chromatin structure. Indeed, the base unit of genetic material compaction is the nucleosome, where DNA is wrapped around an octamer composed of two copies of each histone H2A, H2B, H3 and H4. Different types of enzymes can be recruited to modify chromatin structure: those that use the energy of ATP hydrolysis to alter histone-DNA bonds within the nucleosome, and those that covalently modify certain histone residues, resulting in a more open or more condensed chromatin, respectively more favorable or unfavorable to transcriptional gene expression, depending on the type of modification undergone. The covalent chemical modifications of histones include phosphorylation, methylation and acetylation. The acetylation of core histones is controlled by opposite activity of histone acetyl transferases (HATs) and histone deacetylases (HDACs). Therefore, these enzymes play a key role in the control of gene expression and potentially in the maintenance of tissue homeostasis, including the skin (Kang et al., 2019). To date, most reviews existing in literature have either focused on general epigenetic mechanisms involved in skin homeostasis or when specifically addressing histone acetylation and particularly HDACs, have done it in the context of specific pathologies such as inflammatory or cancer-related diseases (Wu et al., 2025; Rubatto et al., 2023; Palamaris et al., 2022). Despite the value of these contributions, an epidermis-focused analysis of the general functions of HDACs in maintaining this tissue layer is lacking in the literature. This review aims at filling this gap by an overview of the role of HDACs in the biology of the epidermis, their potential involvement in skin diseases and the use of HDACs inhibitors as putative treatment of dermatological conditions with epidermal dysfunctions.

## 2 General information on HDACs

### 2.1 Classification of HDACs

Histone deacetylases play a crucial role in the regulation of gene expression mainly through the removal of acetyl groups from histone proteins, influencing chromatin structure and function (Figure 1a). There are two major families of histone deacetylases: the zinc-dependent HDAC family and the NAD + -dependent sirtuins family.

**FIGURE 1:**
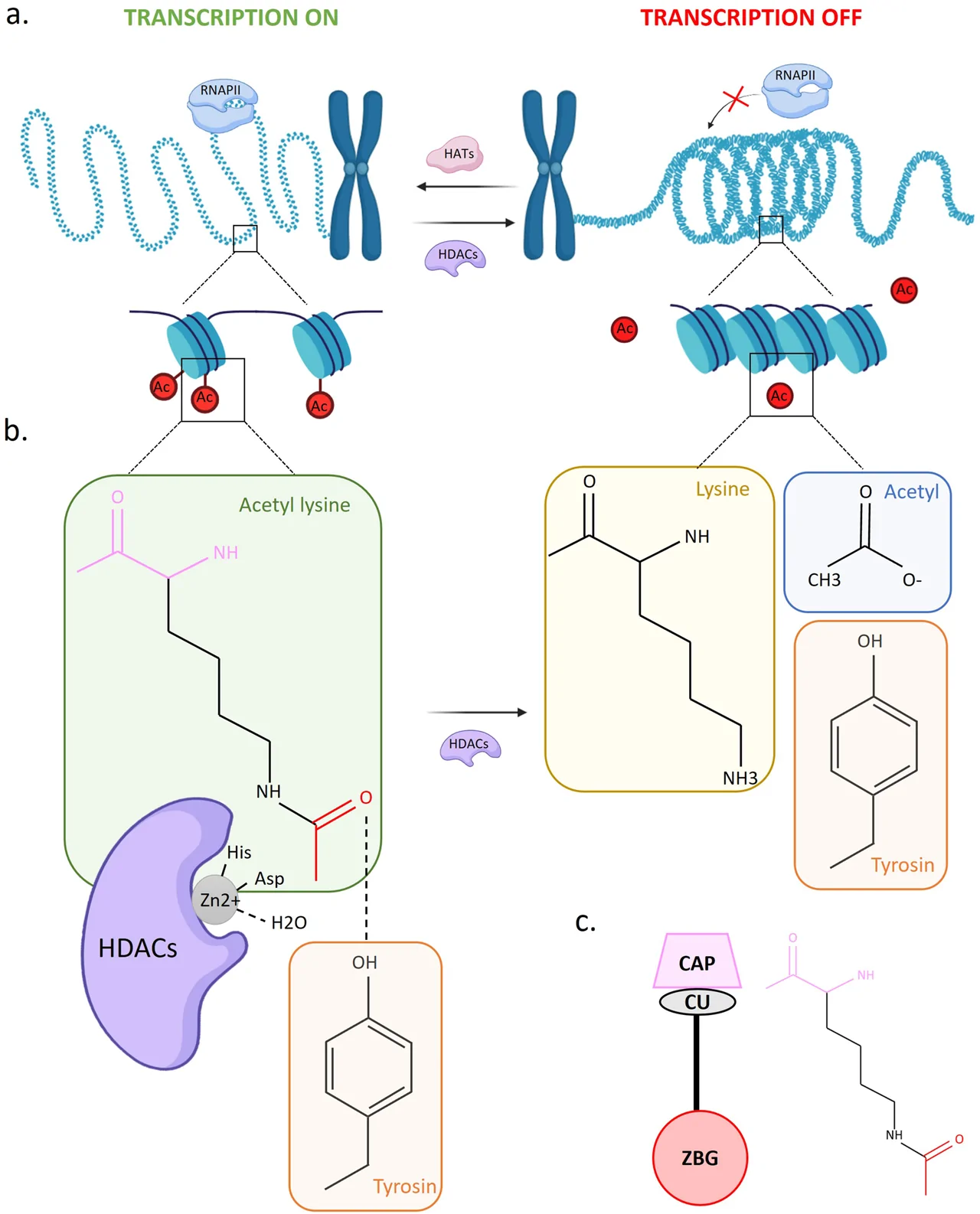
Chromatin state modulation by histone acetylation and HDAC enzymatic mechanism. **(a)** Schematic representation of chromatin dynamics regulated by histone acetylation. Acetylation of histone lysine residues leads to chromatin relaxation, promoting transcription, whereas deacetylation promotes chromatin condensation and gene repression. **(b)** Enzymatic mechanism of histone deacetylases. HDACs catalyze the removal of acetyl groups from acetylated lysine residues, yielding a deacetylated lysine and a free acetate molecule. **(c)** Structural representation of an HDAC inhibitor that mimics polyacetylated lysine, interfering with HDAC activity and maintaining histone acetylation levels.

Following the identification of HDAC1 in 1996 (Taunton et al., 1996), ten other zinc-dependent HDACs were discovered and named according to their order of discovery. These proteins were divided into two classes based on their sequence similarity with yeast histone deacetylases Rpd3 (class I) and Hda1 (class II) (Grozinger et al., 1999). HDAC11 exhibited unique characteristics and is slightly homologous to the existing HDACs (Gao et al., 2002) and was therefore classified as a member of a new and distinct class IV (Gregoretti et al., 2004). Most of the class I HDACs (HDAC1, HDAC2, and HDAC8) are mainly localized in the nuclei of cells, where they play crucial roles in the regulation of gene expression (Ruijter et al., 2003). However, HDAC3 is located in both nucleus and cytoplasm (Waltregny et al., 2004) as Class II (HDAC4, HDAC5, HDAC6, HDAC7, HDAC9, and HDAC10) and class IV (HDAC11) enzymes that exhibit a more diverse subcellular localization (Ruijter et al., 2003).

Sirtuins are NAD + -dependent enzymes that constitute Class III of the histone deacetylases. A total of seven sirtuins have been identified in humans. These sirtuins exhibit diverse subcellular localization: SIRT1 and SIRT2 are found in both the nucleus and cytoplasm, SIRT3 in the nucleus and mitochondria, SIRT4 and SIRT5 in the mitochondria, SIRT6 in the nucleus, and SIRT7 in the nucleolus (Seto and Yoshida, 2014).

### 2.2 Structure of HDACs and mechanisms of histone deacetylation

Early structural insights into HDACs structure, particularly their catalytic core, were obtained from the crystallographic analysis of a histone deacetylase-like protein (HDLP) from the hyperthermophilic bacterium *Aquifex aeolicus*. This bacterial HDLP shares 35.2% sequence identity with human HDAC1 across 375 residues and corresponds to the histone desacetylase catalytic core that is conserved among the HDAC family. The HDLP enzyme core domain has a single-domain structure categorized as an open α/β fold. This structural element creates a deep pocket that is critical for the enzyme’s activity and an internal cavity adjacent to the pocket. The HDLP pocket is lined with hydrophobic and aromatic residues that are identical in HDAC1 and, in this pocket, the zinc ion is positioned by coordination with aspartic acid, histidine residues and water molecule to play a key role in catalysis. Surrounding the pocket, the adjacent internal cavity likely facilitates the movement of reaction products away from the active site (Finnin et al., 1999).

Subsequent studies have explored the structural specificities of each HDAC class and of each HDAC among a same class. Concerning class I HDAC, the first crystal structure of HDAC8 provided more details regarding the specificities of this protein within class I of HDAC in comparison with HDLP (Somoza et al., 2004). In HDAC8, as in HDLP enzyme, aspartic acid and histidine residues (His142, His 143, Asp178, Asp267) are located in the catalytic pocket for zinc coordination (Figure 2a) (Bertrand, 2010). These amino acids are conserved in the catalytic core of most of the zinc-dependent HDACs.

**FIGURE 2:**
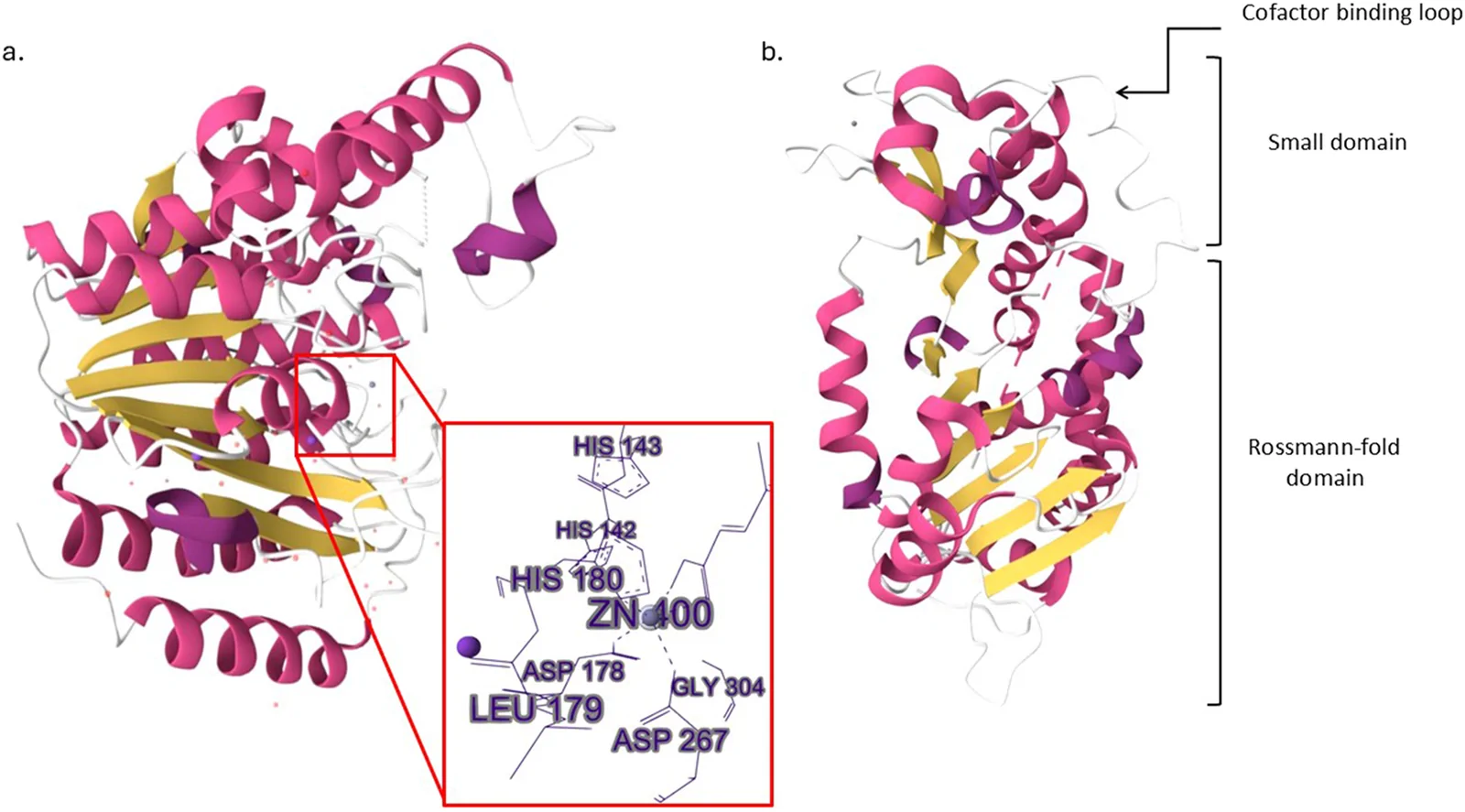
Three dimensional structures of zinc-dependent HDAC and NAD + -dependent SIRT. **(a)** Cartoon crystal structure representation of HDAC8 (PDB ID: 3F0R) colored by secondary structure elements: β-sheets in yellow, α-helices in pink, and loops/unstructured regions in purple. Potassium ions are shown in purple and the catalytic zinc ion in gray. The inset zooms in on the catalytic site show the Zn^2+^ ion coordinated by its key interacting residues. **(b)** Cartoon crystal structure representation of SIRT2 (PDB ID: 1J8F) colored by secondary structure elements: β-sheets in yellow, α-helices in pink, and loops/unstructured regions in purple. Curly brackets indicate the Rossmann-fold domain and the small domain. The cofactor binding loop is indicated. The structural zinc ion is shown in gray.

Crystallographic and biochemical studies of the catalytic domain of class IIa HDACs have revealed their distinct active site configuration. The crystal structure of HDAC4 shows that its global folding is similar to that of HDAC8, containing a Zn2+ ion in the catalytic core and two potassium ions close to the active site. However, HDAC4 lacks the tyrosine hydroxyl group found in HDAC8 that helps for the stabilization of the reaction intermediate. This probably explain why class IIa HDACs have reduce enzymatic activity compared to class I HDACs. In HDAC4, the Zn2+ binding domain is thought to facilitate interactions with the HDAC3/N-CoR complex (Bottomley et al., 2008) and this C-terminal interaction can increase HDAC4 enzymatic activity (Kang et al., 2024).

Class IIb HDACs possess a unique feature within the HDAC family. Indeed, HDAC6 contains two catalytic domains: CD1 and CD2 (Hai and Christianson, 2016) (Table 1). This enzyme has non-histonic protein substrates and it is the CD2 domain that allows the deacetylation of tubulin and Tau protein. However, our knowledge regarding the CD1 domain is still very limited (Osko and Christianson, 2019). The structure of HDAC10 is close to that of HDAC6, since this protein also contains two catalytic sites. The N-terminal catalytic domain of HDAC10 is similar to the deacetylase domain of other known class II HDACs, but the C-terminal catalytic domain does not have enzymatic activity (Cheng et al., 2021).

**TABLE 1:** Structural features of HDAC family members and their localization in epidermal cells.

Family	Enzyme	Structural domain					Localization within the epidermis				
		CD1	CD2	NES	NLS	Zinc finger domain	Basal keratinocytes 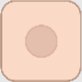	Differentiated keratinocytes 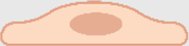	Melanocytes 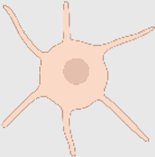	Merkel cells 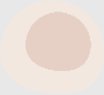	Langherhans cells 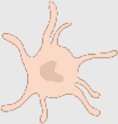
Class I	HDAC1	✓					✓	✓	✓	-	-
	HDAC2	✓					✓	✓	✓	-	-
	HDAC3	✓					✓	✓	-	-	-
	HDAC8	✓					✓	✓	-	-	-
Class IIa	HDAC4	✓		✓	✓		-	✓	-	-	-
	HDAC5	✓		✓	✓		✓	✓	✓	-	-
	HDAC7	✓		✓	✓		✓	✓	-	-	-
	HDAC9	✓		✓	✓		✓	✓	-	-	-
Class IIb	HDAC6	✓	✓	✓	✓	✓	✓	✓	✓	-	-
	HDAC10	✓	✓				✓	✓	-	-	-
Class IV	HDAC11	✓					✓	✓	-	-	-
References		Kang et al. (2024), Cheng et al. (2021), Liu et al. (2012)					Sanford et al. (2016), Winter et al. (2013), Szigety et al. (2020), Sanford et al. (2019), Sawada et al. (2021)		Hu et al. (2020), Woan et al. (2015), Lee et al. (2017), Willis-Martinez et al. (2010), Heppt et al. (2022)	-	Qi et al. (2012)

CD1, CD2: catalytic domains, NES: nuclear export signal, NLS: nuclear localization signal. A tick indicates the presence of the corresponding feature in the corresponding cell or presence of the corresponding HDAC in the indicated epidermal cell type. A dash denotes lack of available information.

Class IV HDACs only consist of HDAC11, the smallest member of the HDAC family (Gao et al., 2002). HDAC11 exhibits a widened catalytic channel, which facilitates the deacetylation of fatty acids (Kutil et al., 2018). HDAC11 also displays a substitution of aspartic acid located in the entrance of the tunnel and conserved in the class I and class II HDACs with asparagine which is probably explaining its notably low catalytic activity (Liu et al., 2023).

Sirtuins, which constitute class III HDACs, have the particularity that they require NAD + for their deacetylation activity, while the other HDAC enzymes depend on zinc. SIRT2 contains a catalytic core with two domains: a large domain that adopts a Rossmann fold, characteristic of NAD(H)/NADP(H)-dependent enzymes, and a smaller domain that contains a helical module and a zinc binding motif and which is crucial for maintaining the structural integrity of the protein (Finnin et al., 2001). These two domains form a pocket in the middle where bind NAD and acetylated peptides (Wu et al., 2022) (Figure 2b).

Despite structural differences existing between HDACs from Class I, II, and IV, their enzymatic mechanism remains very similar. Acetyl-lysine substrate binds to the enzyme’s active site within a channel lined by hydrophobic residues. A key tyrosine (or histidine depending of the HDAC class) residue undergoes a conformational change, which allows the creation of a hydrogen bond with the carbonyl oxygen of the acetyl-lysine. Simultaneously, the zinc ion binds to a water molecule that performs a nucleophile attack on the carbonyl of the acetyl group attached to the lysine producing free lysine and acetate (Figure 1b) (Ho et al., 2020).

In class III HDACs, the catalytic mechanism differs notably due to their dependence on NAD^+^. The catalytic process of Sir2 begins when NAD + binds to the enzyme probably after acetyl-lysine binding. The cleavage of the glycosidic bond in NAD^+^ occurs, leading to the formation of an oxocarbenium ion. The acetylated lysine of the substrate is then positioned within the active site and generates a first intermediate by nucleophile attack on this ion. A second intermediate is produced by deprotonation of the 2′-OH group of ribose. This intermediate undergoes further transformations leading to the formation of the final products including the deacetylated lysine (Sanders et al., 2010).

### 2.3 Expression and distribution of HDACs in the epidermis

Class I HDACs are ubiquitously expressed across all tissues (Haberland et al., 2009). The localization of HDAC isoforms in epidermis cells (keratinocytes, melanocytes, Merkel cells, and Langerhans cells) is presented in Table 1. There is only few data available regarding the absence or presence of each HDAC in healthy melanocytes, Merkel cells and Langerhans cells. Several studies have reported their expression or functional involvement under tumor-related conditions but they do not constitute direct proof of specific HDAC localization in these cells under healthy conditions (Hu et al., 2020; Song et al., 2021; Mazziotta et al., 2022; Kim et al., 2023; Liu et al., 2016; Uzdensky et al., 2014). If we focus on human keratinocytes, all HDACs are expressed at the transcriptional level in this cell type. Among them, HDAC3 transcripts are the most abundantly expressed in both basal and differentiated layers. Transcripts of other HDACs are detected at lower levels in keratinocytes. Some, such as HDAC9, are predominantly expressed in the basal layer, whereas others, including HDAC4, HDAC5, HDAC6, HDAC7, HDAC8, HDAC10, and HDAC11, are primarily expressed in differentiated keratinocytes (Sanford et al., 2016). This differential transcriptional expression suggests that specific HDACs play distinct roles in keratinocyte proliferation and differentiation, reflecting their involvement in skin homeostasis and the potential regulation of epidermal differentiation processes. Some HDACs, such as HDAC1, HDAC8 and HDAC9 have already been detected by immunostaining in human epidermis (Sanford et al., 2016; Shin et al., 2017). However, further protein-level studies are expected to better characterize the localization of different HDACs in epidermal layers.

## 3 HDACs inhibitors

### 3.1 Mechanisms of inhibition

There are numerous chemical HDAC inhibitors, which can be categorized into several classes based on their chemical nature, including hydroxymates, short chain fatty acid, benzamides and cyclic tetrapeptides. Among these, some inhibitors are highly specific, while others act as pan-inhibitors, targeting multiple HDACs. The inhibition mechanism of HDAC inhibitors is mainly characterized by the ability of these compounds to block enzyme activity by binding to the zinc ion located in the catalytic site, thus preventing access to the substrate (Li and Sun, 2019). It has been described that most HDAC inhibitors share a common structure consisting of four key components: a CAP group, a connecting unit, a linker region, and a zinc-binding group (ZBG) (Figure 1c). The ZBG plays a crucial role by chelating the Zn^2+^ ion, which is essential for the enzymatic activity of HDAC and which is located within the enzyme’s active site. The linker region mimics the side chain of N-acetyl-lysine, the natural substrate of HDACs and connects the ZBG to the connecting unit. Finally, the CAP group interacts with the amino acid side chains surrounding the catalytic pocket of the enzyme (Marek et al., 2013). The structural composition of these different components significantly influences the specificity of HDAC inhibitors toward particular HDAC. Variations in the nature of these structural elements impacts the zinc hydroxamate chelation and may confer greater affinity for some HDAC subtypes, highlighting the potential for development of selective HDAC inhibitors in addition to pan-HDAC inhibitors.

### 3.2 Pan-inhibitors and isoforms-specific inhibitors

One of the most common pan-inhibitors is Vorinostat (also known as SAHA), which belongs to the hydroxamic acid class. The inhibition of HDAC catalytic activity by SAHA is mediated by its binding to the zinc ion located in the enzyme’s catalytic domain. Vorinostat functions as an inhibitor of both Class I and Class II HDACs (Wawruszak et al., 2021). It was the first HDAC inhibitor approved by the FDA in 2006 for the treatment of cutaneous T-cell lymphoma (Duvic and Vu, 2007). Another well-known HDAC inhibitor is TSA, which belongs to the hydroxamate family. TSA, originally identified as a fungistatic antibiotic from *Streptomyces* hygroscopicus, was later shown to induce histone hyperacetylation in mammalian cells by inhibiting HDAC activity (Yoshida et al., 1990). Like SAHA, TSA acts non-competitively by mimicking lysine substrates and chelating zinc atom essential for enzyme function (Shankar et al., 2008). TSA exhibits a similar inhibition constant Ki across various HDAC isoforms, suggesting that it is a pan-inhibitor, like vorinostat (Drummond et al., 2005). Belinostat (or PXD101) is an HDAC inhibitor that also belongs to the hydroxamate class and bind to the zinc finger on HDAC (Rossi et al., 2023). This inhibitor is notable for being FDA-approved for the treatment of patients with peripheral T-cell lymphoma (Lee et al., 2015). Panobinostat (LBH589) is an HDAC inhibitor that has also been approved by the FDA for the treatment of patients with multiple myeloma (Laubach et al., 2015). In the context of the skin, few studies exist on this inhibitor, except for a clinical trial investigating its use as a therapy for the treatment of refractory cutaneous T-cell lymphoma (Duvic et al., 2008). Other less commonly used pan-inhibitors include givinostat, resminostat, quisinostat, and abexinostat (Gatla et al., 2019).

Pan-HDAC inhibitors are highly valuable for broad therapeutic applications due to their ability to target multiple HDAC isoforms. However, their clinical application can be limited by toxicity, including off-target and adverse effects such as fatigue, gastrointestinal disturbances and thrombocytopenia which have been reported with the use of Vorinostat, Belinostat and Panobinostat (Liang et al., 2023). In addition, various cardiac abnormalities including ST-T segment abnormalities and QTc interval prolongation of the electrocardiogram also have been observed (Shah, 2019). Therefore, developing isoform-specific HDAC inhibitors is a good therapeutical strategy to attenuate toxicity but also to enable more precise studies of individual HDAC isoforms, helping to clarify their distinct biological functions and roles in various diseases. For example, HDAC6 has been shown to play a crucial role in modulating T-cell receptor signaling. Preclinical studies demonstrated that Ricolinostat and Tubacin, two HDAC6 inhibitors are effective in treating contact hypersensitivity (CHS) in murine models suggesting that HDAC6 could also be a promising therapeutic target for patients with other CD8 T cell–mediated diseases such as vitiligo, graft-versus-host disease (GVHD), and alopecia (Tsuji et al., 2015).

## 4 Physiological roles of HDACs in the epidermis

### 4.1 Cell proliferation and differentiation

Among the physiological roles of HDACs in the epidermis, the regulation of cell proliferation and differentiation have been well described. It has been demonstrated that the use of TSA, a pan-HDAC inhibitor, on human keratinocytes results in a significant reduction of cell proliferation. In parallel, with this alteration of cell proliferation, an increase in the levels of the acetylated form of p53-AcK379 and levels of p21 were observed by immunofluorescence after treatment with TSA. Keratinocytes transduction with shRNAs allowing a p53 and p21 knock-down, followed by their treatment with TSA, restored cell proliferation suggesting that the inhibition of cell proliferation induced by TSA is dependent on the p53 and p21 pathway (LeBoeuf et al., 2010). Another study corroborates this finding and shows that homozygous epidermal co-deletion of HDAC1/2 decreases basal cell proliferation that is rescued by loss of p53 (Zhu et al., 2022). On the other hand, treatment of keratinocytes with butyrate, another HDAC inhibitor, similarly resulted in a significant impairment of cell proliferation, accompanied by a marked reduction in CDK1 transcripts, analogous to the effects observed with TSA (Saunders et al., 1999). Furthermore, another study investigating skin healing mechanisms demonstrated that the use of a SIRT1, 2, and 3 activator, MC2526, accelerates the healing process and enhances keratinocyte proliferation (Spallotta et al., 2013).

Very little is known regarding the role of histone acetylation in the differentiation process of epidermal cells (Leśniak, 2024). It has been demonstrated that knockout of HDAC1 and HDAC2 in embryonic mouse epidermis results in the absence of differentiation markers K10 and loricrin, suggesting a role of HDACs in suprabasal epidermal differentiation (LeBoeuf et al., 2010). Conversely, an increase in the expression of the suprabasal marker involucrin was observed in primary keratinocytes treated with sodium butyrate, suggesting that involucrin may be a target of HDACs and that its expression levels could be regulated by histone acetylation levels (Elder and Zhao, 2002).

### 4.2 Regulation of immune functions

Several studies have demonstrated the involvement of HDACs in skin immune functions through various mechanisms, such as the regulation of macrophages and dendritic cells and the production of pro-inflammatory cytokines, as well as the modulation of T lymphocytes. Beyond their role in histone deacetylation, HDACs target non-histone proteins, including nuclear, cytosolic, and mitochondrial proteins. They can also regulate the acetylation of transcription factors, which impacts their stability, structure, or function (Watson et al., 2024). Among the transcription factors regulated by HDACs, NF-κB, which consists mainly of two subunits: p65 (RELA) and p50 (NFκB1) plays a crucial role in the immune system and is involved in the inflammatory responses mediated by cytokines such as IL-6, IL-8, IL-1β, and GM-CSF. By regulating the acetylation of NF-kB transcription factor, HDAC1 and HDAC2 indirectly influence the transcription of pro-inflammatory cytokines, therefore helping to balance inflammatory responses (Licciardi and Karagiannis, 2012). However, it is important to note that NF-κB acetylation has distinct effects on the expression of NF-κB dependent genes and on its transcriptional activity, depending on the specific target lysine residues. For instance, HDAC3 positively regulates the expression of inflammatory genes by modulating the deacetylation of lysins 122, 123, 314 and 315, while deacetylation at lysine 310 has an inhibitory effect (H et al., 2025). It has also been reported that HDACs inhibition acetylates and activates the transcription factor STAT-3 which induce the transcription of indoleamine 2,3-dioxygenase (IDO), a potent immune-suppressive enzyme in dendritic cells and consequently modulate the function of these cells. Also, since IDO activates Treg cells and blocks their conversion into TH17 cells, it highlights the critical role of HDAC in the regulation of immune response (Sun et al., 2009; Baban et al., 2009). Interestingly, some evidence suggest that HDAC3-deficient macrophages fail to activate half of the inflammatory gene expression upon lipopolysaccharide (LPS) stimulation (Chen et al., 2012). A recent review has highlighted the complexity and contrasting effects of HDACs on cytokines and chemokines regulation. Indeed, HDAC inhibition has been shown to suppress the expression of some cytokines such as IFN-alpha, IFN-gamma and IL-6 and also leads to the reduction of many chemokines of the CCL and CXCL families (CCL3, CCL4, CCL7, CCL8, CCL11, CCL17, CX3CL1, CXCL1 and CXCL6). However, on the other hand, HDAC inhibition appears to increase the expression of CCL20, CXCL8, GM-CSF and has a variable effect on the expression of CCL2, CCL5, CXCL2, CXCL9, CXCL10, CXCL12, IL10 and IL1B. Thus, the variable effect observed suggest that the impact of HDAC inhibition is highly context-dependent (cell type, nature of the inhibitor used, experimental conditions or targeted HDAC-isoform) (Gatla et al., 2019). Together, these findings demonstrate the critical role of HDAC in the regulation of immune functions therefore highlighting their potential involvement in skin pathologies such as psoriasis and atopic dermatitis.

### 4.3 Wound repair and healing

The involvement of HDACs in immune responses and cell proliferation suggests that these enzymes may also play a crucial role in wound healing and tissue repair processes. Studies have demonstrated that the use of SIRT activators promotes skin repair and accelerates wound healing by enhancing keratinocyte proliferation through nitric oxide (NO)-dependent mechanisms. Although HDACs are often associated with cell proliferation, this study demonstrates that the use of HDAC inhibitors, such as TSA, paradoxically also accelerates skin healing. This contradiction can be explained by a NO-dependent cross-talk between class I HDACs and sirtuins: sirtuin activation promotes eNOS deacetylation and activation, which increases NO production, resulting in the S-nitrosylation and inhibition of HDAC2. This in turn, leads to the release of repair genes like keratinocyte growth factor 2, fibroblast growth factor 10 and insulin-like growth factor. Consequently, the use of chemical HDACs inhibitors alone, can promote wound healing (Spallotta et al., 2013). Furthermore, another study explored the impact of HDAC inhibition on the plasticity of macrophages derived from bone marrow myeloid progenitors (BMMP). After treatment with HDAC inhibitors, BMMPs differentiate into elongated macrophages with a reparative phenotype, therefore facilitating wound closure. This effect is linked to chromatin remodeling, which controls the expression of genes involved in inflammation and tissue repair. *In vivo*, skin wound treated with HDAC inhibitor showed accelerated wound closure (Cabanel et al., 2019). Another study investigated the role of HDAC in the context of diabetic wounds, where excessive activation of inflammasomes and high production of pro-inflammatory cytokines, such as IL-1β in macrophages, impair the healing process. They demonstrated that the HDAC inhibitor TSA, promotes wound healing in diabetic mice by regulating the expression of IL-1β and IL10. TSA reduced the infiltration of neutrophils, T lymphocytes, and macrophages during the wound healing process. The topical application of HDAC inhibitors on diabetic wounds also enhances collagen formation, angiogenesis, and reduces inflammation, thereby accelerating wound closure (Karnam et al., 2020). Regarding angiogenesis, several studies have highlighted the crucial role of HDACs in this key healing process but their functions appear to be contradictory depending on the isoform and pathological context. For example, HDAC1 and HDAC3 can promote vessel regeneration by inhibiting the expression of anti-angiogenic genes, while HDAC5 has an anti-angiogenic effect, limiting the migration and sprouting of endothelial cells (Zhang et al., 2025). Thus, although HDACs are traditionally associated with cell proliferation support, their inhibition may paradoxically promote wound healing by modeling inflammatory, reparative responses and angiogenesis. HDACs are also able to regulate signaling pathways that are crucial for wound healing, such as the Wnt/β-catenin, Notch, and HIF-1α/VEGF pathways (Zhang et al., 2025). However, some effects appear to be isoforms and context dependent, highlighting the complexity of HDAC functions in wound repair. Fine-tuned regulation of their activity allows cells to shifts from an inflammatory to a regenerative phenotype but further studies are required to clarify the role of HDAC in this process.

## 5 HDACs and epidermal pathologies

### 5.1 Psoriasis and atopic dermatitis

Psoriasis is a chronic inflammatory skin disorder characterized by an excessive Th-17 mediated immune response due to exaggerated cytokine signaling. In psoriasis, chronic inflammation ultimately leads to keratinocytes hyperproliferation and abnormal differentiation with dysregulated apoptosis. Several HDACs exhibit modified expression in psoriasis lesions. This is the case of HDAC3 expression which has been shown to be significantly elevated in human psoriasis lesions as well as in *vitro* and *in vivo* models of psoriasis. In an inflammatory context, HDAC3 promotes oxidative stress and disrupts mitochondrial integrity leading to the release of mitochondrial DNA. This, in turn, activates the cGAS-STING pathway in keratinocytes, a pathway found to be upregulated in psoriatic lesions and IMQ-induced psoriasis-like mouse models and that is known to promote the expression of proinflammatory cytokines (Zeng et al., 2025) (Figure 3). HDAC1 transcripts were over-expressed in most of the affected psoriatic skin samples compared to unaffected psoriatic skin and healthy (Tovar‐Castillo et al., 2007). While HDAC1 and HDAC2 show normal expression in non-lesional psoriatic skin, the expression of their repressor, SPHK2, is altered and leads to impaired differentiation of Th17 lymphocytes in psoriasis. There is also an alteration in the expression levels of some members of HDAC1-2 protein complexes affecting the function of the NURD, SHIP, and SIN3 complexes. The NURD complex mainly plays a key role in controlling T cell development and their cell cycle progression but also progenitor cell maintenance. More specifically, CHD4 contained in the NURD complex has an altered expression in non-lesional psoriatic skin, thus impacting Th2 cell differentiation and CD8^+^ T-cell infiltration. The SHIP complex and more specifically HSPA2, a member of this complex contributes to the early differentiation of keratinocytes and the establishment of epidermis stratification. Finally, SIN3 complex whose expression is altered in non-lesional skin of psoriasis regulates the development of T cells and in particular the differentiation of TH17 and the establishment of their inflammatory potential (Romhányi et al., 2023). Modulation of some HDACs expression is also observed in non-lesional psoriatic skin compared to normal skin. Altered expression of HDAC8 in non-lesional psoriatic skin modulates keratinocyte tolerance to certain immune stimuli and increases T cell infiltration (Romhányi et al., 2023). HDAC4, HDAC5, and HDAC6 also show altered expression in non-lesional psoriatic skin compared to healthy skin. HDAC4 seems to exert both pro- and anti-inflammatory effects depending on its target: when acting as a transcriptional repressor of NF-κB, it decreases pro-inflammatory cytokine production, whereas its indirect activation of Foxo3a, a transcription factor, leads to increased inflammation in vascular endothelial cells (Romhányi et al., 2023; Yang et al., 2018). Moreover, Foxp3 is a crucial regulator of Treg cells differentiation and function. A defect in the expression of Foxp3 can promote the onset of inflammatory and autoimmune diseases such as psoriasis in which T regs are dysfunctional and cannot suppress the activity of Th17 cells (Kanda et al., 2021). It has been demonstrated that HDACs are involved in the stability and expression of Foxp3 since HDAC10 can inhibit Foxp3, SIRT2 and SIRT4 downregulate its protein expression and HDAC3, HDAC6, HDAC9, HDAC11, and SIRT1 destabilize Foxp3 through deacetylation, thus making it more sensible to degradation by the proteasome (Von Knethen et al., 2020). Interestingly, it had been demonstrated that HDAC5 can regulate the differentiation of CD4^+^ T cells into Tregs and cytokine production from CD8^+^ T cells, therefore aggravating inflammation. Indeed, in mice, CD4 (+) T-cells without HDAC5 convert poorly to Tregs and CD8 (+) T cells produce less cytokine IFN-γ (Romhányi et al., 2023; Xiao et al., 2016) (Figure 3). Class III and IV HDACs, SIRT5, SIRT6, and HDAC11 show abnormal expression in non-lesional skin. Both SIRT5 and SIRT6 negatively regulate inflammation, and HDAC11 plays a crucial role in the regulation of immune and inflammatory responses, notably through T cell activation (Romhányi et al., 2023). Altogether, these data reveal a complex role of HDACs in inducing pro inflammatory pathways which can lead to the development of psoriasis, acting indirectly through activation of the cGAS-STING pathway or directly by regulating Foxp3, the critical regulator of Treg conversion into Th17 cells leading to exacerbated immune response (Figure 3).

**FIGURE 3:**
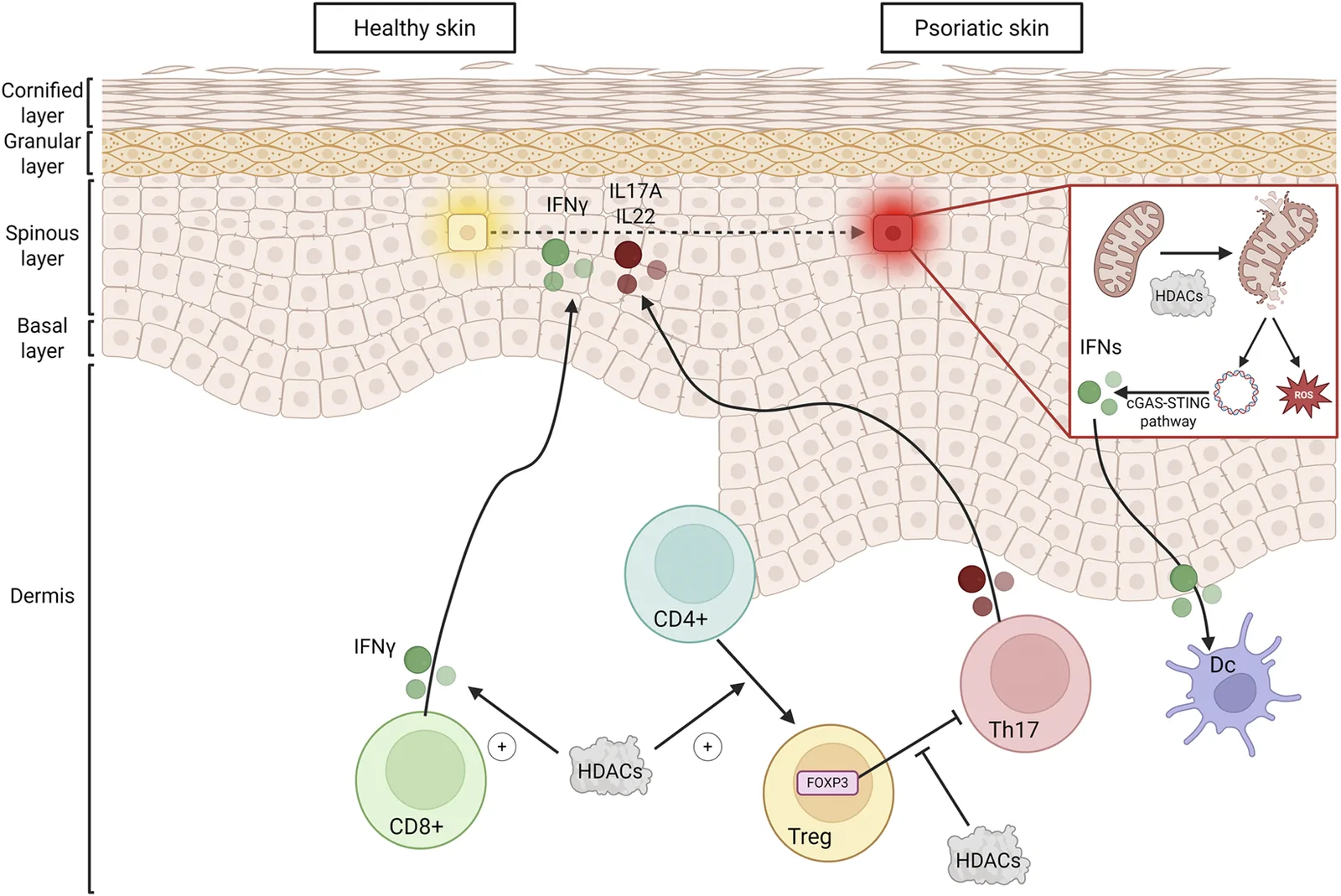
Schematic representation of healthy and psoriatic skin and the role of HDACs in inflammatory processes.

Several studies have explored the use of HDAC inhibitors as potential treatments for this chronic inflammatory skin disorder. The use of HDAC inhibitors, such as the pan-inhibitor trichostatin A, promotes increased Foxp3 expression and contributes to the post-transcriptional stabilization of Foxp3, thereby preventing the conversion of Treg cells into Th17 cells (Kanda et al., 2021; Jorn Bovenschen et al., 2011; Koenen et al., 2008). The application of Entinostat, a specific HDAC1 inhibitor, on imiquimod mouse models improved the psoriasis-induced inflammation through the reduction of infiltrating IL-17A+ γδT cells into the skin, cells that are responsible of IL-17A production, the major cytokine responsible of psoriasis physiopathology but also through the inhibition of Th17 cell generation (Jiang et al., 2023; Zhang et al., 2024). Another study has demonstrated that piperlongumine (PPL) could act as an antiproliferative and anti-inflammatory agent, particularly through the inhibition of HDAC enzyme activity and the reduction of their expression in macrophages and skin tissues. Indeed, PPL treatment was shown to prevent the nuclear translocation of the p65-HDAC3 complex, thereby interfering with the transcription of inflammatory genes (Thatikonda et al., 2020). The use of HDAC inhibitor vorinostat, could be an effective treatment for psoriasis, primarily by inhibiting keratinocyte proliferation and promoting their differentiation and apoptosis. Indeed, vorinostat treatment in a mouse xenograft model of psoriasis result in a reduction of disease phenotype with decreased epidermal thickness and reduced keratinocyte proliferation (Samuelov et al., 2022). Application of remetinostat, another HDAC inhibitor, in imiquimod-induced mouse models also has been shown to improve psoriasis symptoms, primarily through the inhibition of CD86 expression on skin dendritic cells, as well as keratinocyte differentiation and inflammation (Jin et al., 2024). Finally, it had been recently demonstrated that the use of CS1, a novel specific HDAC6 inhibitor, attenuate LPS-induced inflammation *in vitro* and improve IMQ-induced psioriasis-like inflammation in mice thanks to the inhibition of keratinocyte hyperproliferation through AKT pathway blocking but also the reduction of inflammatory cells infiltration through MAPK and STAT3 pathway inhibition (Cao et al., 2025). All these findings suggest that HDAC inhibitors are good candidates for the development of anti-psoriasis therapies.

Atopic dermatitis (AD) is a common inflammatory skin disease characterized by intensive itch, eczema-like eruptions, and dry skin (Sroka-Tomaszewska and Trzeciak, 2021). Sebum secretion significantly influences the composition of the skin microbiome. In patients with AD, sebaceous glands are often smaller and fewer and produce less sebum, which affects skin hydration and barrier function. Abundant sebum production provides the skin microbiota with the substrates necessary for lipid metabolism, leading to the production of short-chain fatty acids (SCFAs). Among SCFAs, propionate appears to play a crucial role in AD, as its concentrations on the skin surface are relatively higher than those of other SCFAs, and significantly lower in AD patients than in healthy individuals. Propionate application on the skin of mice with MC903-induced AD-like dermatitis was found to reduce skin inflammation through the decrease of IL-33 production in keratinocytes. This effect is mediated via the inhibition of HDAC2 and HDAC3, which regulate the aryl hydrocarbon receptor (AhR) (Qiu et al., 2022). A recent study focused on the effects of particulate matter (PM) in AD via the alteration of filaggrin and HDAC expression. It has been demonstrated that exposure to PM can worsen AD, one of the major causes of which is the alteration of the skin barrier, often associated with loss or dysfunction of filaggrin. In AD-induced HEK cells, the transcript levels of HDAC3 and HDAC6 were increased, and this increase was even more pronounced after exposure to PM. Conversely, the expression of filaggrin was decreased in this AD model, and PM exposition accentuated this decline. Similar observations on the modulation of filaggrin and HDAC expression have also been reported in murine AD-like models exposed or not to PM. *In vitro*, treatment with TSA decreased HDAC3/6 expression and restored filaggrin levels. These results suggest that inhibition of HDAC could attenuate PM-induced epigenetic dysregulation in DA. However, to determine if HDACs directly regulate the filaggrin promoter, further studies, such as chromatin immunoprecipitation (ChIP) are required (Roh et al., 2025). Belinostat has been also studied as a potential therapy for atopic dermatitis. Indeed, a study shows that miR-335 is essential for maintaining epidermal homeostasis, and its loss in the lesional skin of atopic dermatitis patients leads to impaired keratinocyte differentiation. This miR is epigenetically regulated by HDACs, and the use of the pan-HDAC inhibitor belinostat restores miR-335 expression, thereby enhancing keratinocyte differentiation and correcting skin barrier defects associated with AD (Liew et al., 2020).

### 5.2 Skin cancer

There are three main types of skin cancer involving epidermal cells: basal cell carcinoma (BCC), squamous cell carcinoma (SCC), and cutaneous melanoma (Gordon, 2013). Carcinomas are the most common forms of skin cancer. BCC is generally less invasive and therefore more treatable than SSC. Melanomas, which develop from melanocytes, represent the most aggressive form of skin cancer and can spread to other organs. Due to the significant involvement of histone deacetylases in cellular proliferation mechanisms, these proteins have generated an increasing interest from researchers as targets for the development of anti-cancer therapies. Indeed, it had been shown that aberrant expression of HDAC level have been observed in many cancers including cutaneous melanoma (Sakuma et al., 2006; Hornig et al., 2016). Studies have demonstrated that HDAC inhibition leads to a reduction in cancer cells proliferation and induces cell death, mainly through the transcriptional activation of p21 and p53, the suppression of cyclins, and enhanced interactions between tumor cells antigens and adaptive immune cells (Palamaris et al., 2022). In melanoma, a decreased expression of some tumor suppressor genes, such as CDKN1A, due to the deacetylation of some lysine residues in histones by HDACs has been observed. However, it has been demonstrated that the use of TSA, results in an increase of p21^cip1^ encoded by CDKN1A, suggesting that this inhibitor could be used as a therapy to restore the expression levels of tumor suppressor genes (Sarkar et al., 2015). It has also been shown that HDAC inhibition by TSA in melanocytes induces an antiproliferative effect through the inhibition of Tle3 activity, a mitogenic factor whose expression is elevated in melanoma patients and plays a role in melanoma cell proliferation (Ogawa et al., 2019). The HDAC inhibitor S-8 also appears to be another promising therapeutic candidate for treating melanoma. Treatment of highly metastatic human A375 melanoma cells led to histone H3/H4 and α-tubulin acetylation, cell cycle arrest at G0/G1 and G2/M via increased levels of p21, enhanced apoptosis, reduced motility, invasiveness and pro angiogenic potential (Balliu et al., 2015). It has also been demonstrated that the use of HDAC inhibitors can up-regulate antigen expression or alter antigen presentation in melanoma, thereby increasing their immunogenicity through increased expression of CD25, CD40 or CD80 (Hornig et al., 2016). One study reported an increase in the expression of PD-L1 *in vivo* and *in vitro* in melanoma cells following HDAC inhibitor treatment, due to enhanced histone acetylation of the PD-L1 promoter region. In the context of cancer, expression of PD-L1 impairs tumor reactive T-cells as it activates the inhibitory regulatory pathway PD-1 in T cells and allows immune escape. Even if PD-L1 negatively impacts the immune response against tumors, the upregulation of PD-L1 induced by HDAC inhibitors, when combined with a T cell PD-1 receptor inhibitor, resulted in slower tumor progression and increased survival in the B16F10 melanoma murine model by counteracting the interaction between PD-L1 and the PD-1 receptor on T cells, which otherwise leads to tumor tolerance (Woods et al., 2015). In contrast, another study demonstrated that chemical or genetic inhibition of HDAC6 leads to a decrease in PD-L1 expression, resulting in reduced tumor growth, illustrating the particular role of this HDAC in PD-L1 regulation (Lienlaf et al., 2016). An important proportion of melanomas are characterized by mutations in the BRAF gene that lead to activation of the MAPK/ERK mitogenic pathway and result in increased cell proliferation and more aggressive melanoma. This oncogene is therefore a key target for anticancer therapies. The use of pan-HDAC inhibitors, such as SAHA and givinostat, on melanoma cells carrying the oncogenic BRAF V600E mutation resulted in a reduction of mutated BRAF protein levels and a decrease in melanoma cell viability. Additionally, givinostat was shown to induce a shift from autophagy to classical apoptosis. For these reasons, this HDAC inhibitor represents a promising candidate for melanoma therapies (Celesia et al., 2022). Another study has shown that HDAC inhibitors can be used as combinatorial therapies for melanoma. RRM2 is a binding partner and target of USP7. This cell cycle regulated enzymes is frequently deregulated in cancer. Inhibiting USP7 reduces RRM2 levels, decreasing the proliferation of melanoma cells. The disruption of USP7/RRM2 using a USP7 inhibitor (P5091) therefore prevents tumor progression and induces cancer cells senescence. The administration of the HDAC/LSD1 inhibitor domatinostat, coupled with P5091 treatment, induced an increased acetylation of histone H3, a p53 activation, and an increased expression of gH2AX, promoting the elimination of senescent cells. The combined use of P5091 with other HDAC inhibitors, such as vorinostat and belinostat, showed similar efficacy (Granieri et al., 2022). In another study, the use of the HDAC inhibitor Valproic Acid, led to an increase in the sensitivity of human melanoma cells to apoptosis after treatment with Dacarbazine and PARP inhibitors, enhancing the effectiveness of these two treatments. An increase in DNA double-strand breaks (DSBs) and a reduction in melanoma cell survival and proliferation were also observed with this combinatorial therapy. This therapy’s effectiveness could be explained by the fact that one mechanism by which tumor cells resist PARP inhibitors is through the activation of DNA repair pathway involving homologous recombination (HR). HDACs promote HR repair of DSBs contributing to tumor cell resistance, hence the need to use HDAC inhibitors (Drzewiecka et al., 2023). Another study revealed that SIRT2 is an important regulator in melanoma cells, playing a role in mobility, cell proliferation and treatment resistance. Reducing SIRT2 levels alters the expression of tyrosine kinase receptors and increases the sensitivity of melanoma cells to the multikinase inhibitor dasatinib (Karwaciak et al., 2019).

To conclude, HDACs play a pivotal role in the progression of skin cancers through multiple mechanisms. Among these, HDACs influence gene expression by modulating histone acetylation, which can lead to the deregulation of tumor suppressor genes such as p21, p53, and CDKN1A. Additionally, HDACs can modulate oncogenic and immune signaling pathways, as well as the balance between autophagy and apoptosis. Modulation of HDAC expression is also a good strategy to overcome chemoresistance observed in melanomas in response to therapies.

## 6 Animal models of HDAC knockout: insights into skin biology and disease

The roles of HDAC have been explored through deletion of their encoding genes in mouse skin. The main studies reporting conditional deletions of Hdac1, Hdac2 and Hdac3 in the murine epidermis reveal variable phenotypes depending on the target gene and the context of the deletion (Table 2). Overall, Hdac1^Δ/Δ^Hdac2^Δ/Δ^ deletion results in non-viable animals with major alterations in basal proliferation, increased apoptosis and disorganized differentiation, whereas simple KO Hdac1^Δ/Δ^ or Hdac2^Δ/Δ^ only induce few or no abnormalities, highlighting the functional redundancy between HDAC1 and HDAC2, although some contradictions exist in the literature. In contrast, Hdac3^Δ/Δ^ deletion during embryonic development causes impaired skin barrier function, illustrating a distinct role for this enzyme in epidermal morphogenesis. The divergencies observed in literature concerning animal models of HDAC KO can be attributed to several experimental factors. First, the mouse strains used can differ, LeBoeuf et al. used a multiple background strain while Hughes et al. (2014) worked with C57BL/6J mice, in which skin phenotypes can manifest differently (Hughes et al., 2014). Secondly, the promoter used to target keratinocytes influences the specificity and timing of Cre recombinase expression which can strongly modulate the severity and nature of the phenotype observed. Finally, variables such as the sex and age of the animals can also influence epidermal renewal dynamics and stress response, contributing to inter-study variability.

**TABLE 2:** Summary of cutaneous phenotypes resulting from targeted epidermal deletion of HDAC family members in mice.

Model used	Genotype	Main cutaneous phenotype	References
Individual and combined knockout of Hdac1 and Hdac2 using K14-Cre	Hdac1^Δ/Δ^	normal epidermal development	LeBoeuf et al. (2010)
	Hdac2^Δ/Δ^	normal epidermal development	
	Hdac1^Δ/Δ^ Hdac2^Δ/Δ^	no viable (died perinatally), thin and smooth skin, no epidermal stratification, no hair follicle development, failure of epidermal differentiation, proliferation defect	
	Hdac1^Δ/Δ^ Hdac2^Δ/+^	normal epidermal development	
	Hdac1^Δ/+^ Hdac2^Δ/Δ^	normal epidermal development	
Individual and combined knockout of Hdac1 and Hdac2 using K5-Cre	Hdac1^Δ/Δ^	normal epidermal development	Winter et al. (2013)
	Hdac2^Δ/Δ^	normal epidermal development	
	Hdac1^Δ/Δ^ Hdac2^Δ/Δ^	no viable	
	Hdac1^Δ/Δ^ Hdac2^Δ/+^	alopecia, hyperkeratosis, hyperproliferation and spontaneous tumor formation, functional epidermal barrier	
	Hdac1^Δ/+^ Hdac2^Δ/Δ^	normal proliferation and differentiation	
Individual and combined knockout of Hdac1 and Hdac2 using K14-Cre, male, 9 days to 6 months	Hdac1^Δ/Δ^	epidermal thickening, hyperkeratosis, and increased epithelial apoptosis, alopecia, abnormal hair morphology and abnormal claw pigmentation	Hughes et al. (2014)
	Hdac1^Δ/Δ^ Hdac2^Δ/+^	Same phenotype as Hdac1^Δ/Δ^ but more obvious	
Doxycycline-inducible individual and combined knockout of Hdac1 and Hdac2 in K5 promoter–active basal cells	Hdac1^Δ/Δ^ Hdac2^Δ/Δ^	epidermal thickness, reduce basal cell proliferation, increased apoptosis, inappropriate differentiation	Zhu et al. (2022)
	Hdac1^Δ/Δ^ Hdac2^Δ/+^	epidermal thickness and proliferation were not significantly altered	
	Hdac1^Δ/+^ Hdac2^Δ/Δ^	epidermal thickness and proliferation were not significantly altered	
	Hdac1^Δ/Δ^ Hdac2^Δ/Δ^ P53^Δ/Δ^	Rescue of the reduced proliferation phenotype	
Doxycycline-inducible deletion of Hdac3 in embryonic K5 promoter–active epidermis	Hdac3^Δ/Δ^	erythematous, tight skin, epidermal barrier defect, rough and fragile cornified envelopes	Szigety et al. (2020)

## 7 Discussion

HDACs play an important role in skin epigenetic regulation, influencing essential processes such as differentiation, cell proliferation, immune response, and wound healing. Their involvement in maintaining skin homeostasis, as well as their contribution to the development of various skin pathologies, highlight the complexity of their regulation and the need for precise control of their activity. Insights into their specific functions in epidermis have been gained through conditional HDAC knockout animal models, even if relatively few studies in this area have been conducted. Moreover, conditional deletion studies in mouse models sometimes report divergent results probably due to compensatory phenomena, such as the activation of other HDACs or regulation of other epigenetic modulators including HATs. Additionally, the use of HDAC inhibitors has emerged as a valuable approach to elucidate the biological roles of these enzymes and are good therapeutic strategy to treat cutaneous T-cell lymphoma, atopic dermatitis, and psoriasis. However, the low specificity of available inhibitors which target simultaneously several HDACs makes it difficult to distinguish between effects related to the inhibition of a specific HDAC and those resulting from a combined action of HDACs. Also, off-target effects of inhibitors raise the possibility that some effects attributed to HDAC are actually indirect effects. Further research on the structure of each HDAC could allow the development of more selective inhibitors.

To conclude, despite recent advances, our knowledge concerning the precise role of HDACs in epidermal differentiation, inflammatory regulation and genes directly targeted by HDACs in keratinocytes is still limited. Future epigenetic studies will benefit from recent methodological advances. Genome-wide mapping of HDACs and HATs during epidermal differentiation and wound healing would be very useful to clarify the impact of these epigenetic regulations on key processes of epidermal homeostasis. Single-cell chromatin profiling and spatial epigenomic approaches could provide information on the distinct epigenetic states of each epidermal cell type and enable the mapping of chromatin landscapes across epidermal layers and under physiological or pathological contexts. Finally, interactions between the skin microbiota and the epidermis already known to influence histone modifications and HDAC activity justify further study to clarify their mechanisms, their implications for skin disease and to pave the way for combined therapeutic approaches (Szabó et al., 2025).
